# Iatrogenic Left Main Coronary Artery Dissection With Retrograde Aortic Extension During Complex Percutaneous Coronary Intervention in a Left‐Dominant System: A Case Report

**DOI:** 10.1002/ccr3.73145

**Published:** 2026-07-16

**Authors:** Ghulam Abbas Shaikh, Samina Yaqoob, Ali Aamir, Zahra Anas, Aniqa Dejwani, Atiqullah Sadaqat

**Affiliations:** ^1^ Department of Cardiology Dr Ruth KM Pfau Civil Hospital Karachi Pakistan; ^2^ Department of Internal Medicine Dr Ruth KM Pfau Civil Hospital Karachi Pakistan; ^3^ Department of Internal Medicine DOW University of Health Sciences Karachi Pakistan; ^4^ Department of Curative Medicine Kateb University Kabul Afghanistan

**Keywords:** coronary artery disease, coronary artery dissection, iatrogenic disease, percutaneous coronary intervention

## Abstract

In iatrogenic left main coronary artery dissection, especially with aortic extension, rapid recognition, and immediate, well‐planned percutaneous coronary intervention to seal the entry point is critical to restore flow and prevent collapse, with success hinging on a meticulous technique in high‐risk anatomy and coordinated multidisciplinary care with close follow‐up.

## Graphical abstract

Clinical course of catheter‐induced left main coronary artery dissection during complex percutaneous coronary intervention (PCI) for heavily calcified left main bifurcation disease. Following iatrogenic ostial left main dissection with limited retrograde extension into the aortic root, immediate haemodynamic stabilisation and bailout PCI were performed using sequential drug‐eluting stent implantation, proximal optimisation technique (POT), kissing baloon inflation (KBI), and final POT. The strategy successfully sealed the dissection, restored coronary perfusion, and was associated with no progression on 24 h CT angiography and an excellent short‐term clinical outcome.

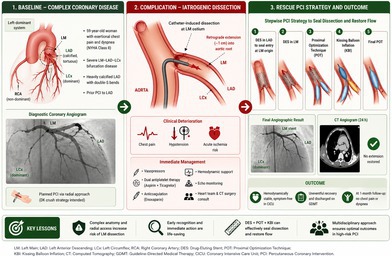

## 1 Introduction

Coronary artery disease (CAD) continues to be a major cause of mortality globally, accounting for approximately 7 million (20%) deaths and 129 million disability‐adjusted life years each year, significantly impacting global healthcare costs and disability [1]. CAD is a complex condition that evolves through changing stages and appears as either acute or chronic coronary syndromes [2]. The landscape of CAD management has been profoundly transformed by advances in percutaneous coronary intervention (PCI), which is now the preferred revascularisation strategy for many patients with acute and chronic coronary syndromes [3]. Left main coronary artery (LMCA) disease has a very high risk of mortality and morbidity, as it provides almost 84% of the left ventricle's blood supply (left dominant system) [4], and PCI is considered a reasonable alternative in select patients with less complex anatomy [3]. However, despite its high procedural safety profile, PCI is not without risks. Among its most feared complications is iatrogenic left main coronary artery dissection, especially when it extends retrogradely into the aortic root, creating an acute aortocoronary dissection [5].

Iatrogenic LMCA dissection, defined as separation of the vessel wall typically caused by manipulation of a guiding catheter or coronary interventional procedures, is rare, with a reported incidence of less than 0.1% during PCI. Its clinical impact is profound, with a significantly increased risk of abrupt vessel closure, aortic involvement, sudden haemodynamic collapse (15.6% of cases) and mortality (5.9%) [6, 7]. LMCA dissection may remain asymptomatic or, by compromising the true lumen, may result in ischaemia or infarction with varied manifestations [6]. Clinically, the onset of iatrogenic LMCA dissection is more often acute, with new or recurrent chest pain, hypotension, arrhythmias, and sudden loss of coronary flow, necessitating prompt haemodynamic support and immediate action in the catheterisation laboratory [7]. Dilatation of the vessel and persistent contrast staining are the immediate clues for diagnosis (6), and acute management involves rapid sealing of the dissection entry point, typically by deploying stents to reestablish vessel integrity and prevent further extension into the aorta [8]. The absence of robust, evidence‐based guidelines for these rare events underscores the crucial role of operator judgment, situational awareness, and multidisciplinary collaboration in their management.

Herein, we present a rare case of iatrogenic LMCA dissection with retrograde extension into the aorta during complex PCI for severe CAD in a left‐dominant system. In this report, we aim to highlight the clinical features, management strategies, and outcomes associated with this life‐threatening complication, contributing to the limited but vital literature that informs and guides interventional practices.

## 2 Case History/Examination

### 2.1 Case History

A 59‐year‐old woman with a history of hypertension presented with a 1‐week history of exertional chest pain, shortness of breath (YHA Class II), and palpitations. The chest pain ranged from dull to severe and was described as heaviness radiating to the left arm and shoulder. It was precipitated by physical activity, such as climbing stairs or walking for 10–15 min, and relieved by resting. The pain was non‐positional and not related to movement. The episodes were associated with shortness of breath.

The patient also reported recurrent syncopal episodes over the preceding 2 years, occurring five to six times and lasting approximately 15–20 min each. There was no history of pedal oedema, abdominal distention, cough, or fever.

Her medical history was significant for recently diagnosed hypertension (10 days prior), ongoing treatment for *Helicobacter pylori* infection, and hepatitis C diagnosed 8 months earlier, for which she had completed a 3‐month treatment course. She had previously undergone PCI of the left anterior descending (LAD) artery via the radial approach. The patient had no family history of cardiac disease.

Her personal history revealed anorexia, unintentional weight loss of 7 kg, insomnia, and chronic betel (areca) nut dependence for over two decades. The systemic review was positive for unilateral headaches, photosensitivity, wheezing, intermittent arthralgia, oral ulcers, black stools, constipation, dyspepsia, heartburn, dysuria, and weak urinary stream. No upper respiratory tract symptoms or focal neurological deficits were observed.

### 2.2 Examination

On examination, the patient was haemodynamically stable and afebrile. Cardiovascular examination revealed a regular heart rhythm, without murmurs or additional heart sounds. Electrocardiography (ECG) revealed a sinus rhythm without acute ischaemic changes.

Laboratory investigations showed normal complete blood count, electrolyte levels, renal function, and liver function. Cardiac biomarkers demonstrated an elevated troponin I level of 0.1, suggesting myocardial injury. The lipid profile revealed mildly elevated total cholesterol and reduced high‐density lipoprotein (HDL) levels.

Transthoracic echocardiography revealed a small akinetic apical segment of the left ventricle with preserved global systolic function (ejection fraction: 55%). Mild mitral and tricuspid regurgitation was noted without pericardial effusion or structural valvular abnormalities. The right ventricular function was normal, and the inferior vena cava (IVC) showed normal respiratory variation.

## 3 Methods

### 3.1 Differential Diagnosis

Based on the presenting symptoms and initial evaluation, the differential diagnoses included acute coronary syndrome (ACS), stable angina with progression of coronary artery disease, in‐stent restenosis, arrhythmia‐related syncope, aortic pathology including aortic dissection, vasovagal syncope, and structural heart disease.

### 3.2 Investigations

Coronary angiography via the radial approach was performed for further evaluations. Angiography revealed (Figure 1):

- Severe proximal stenosis of the LAD involving the ostium of the first diagonal branch (D1)
- Corresponding stenosis in the left circumflex artery (LCx) involving the ostium of the first obtuse marginal branch (OM1)
- Dominant LCx circulation with a nondominant right coronary artery (RCA)

**FIGURE 1:**
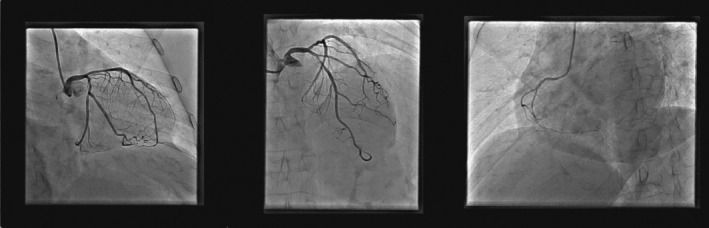
Selective left heart catheterisation performed via the radial approach, demonstrating severe calcified stenosis of the mid left anterior descending (LAD) artery with associated mild proximal disease of the left circumflex (LCx) artery. The right coronary artery (RCA) was nondominant and free of significant obstructive disease. These angiographic findings prompted planned percutaneous coronary intervention (PCI) to the LAD artery.

The LAD was heavily calcified (grade 3) with double S‐bends, making device navigation challenging. During attempted PCI using a planned double kissing crush (DK crush) strategy for the left main (LM)–LAD–LCx bifurcation lesion, device delivery through the radial route failed because of severe tortuosity and calcification.

An iatrogenic dissection subsequently developed at the ostium of the LM coronary artery with retrograde extension (~1 cm) toward the aortic root, involving both the LAD and LCx (Figure 2). The dissection appeared to be contained without evidence of vessel perforation.

**FIGURE 2:**
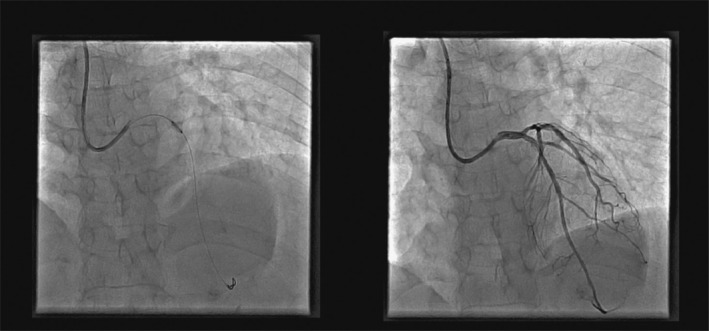
Guidewire successfully advanced across the lesion, with initial vessel preparation performed using a 2.0 × 10 mm semi‐compliant (SC) balloon.

Following the event, the patient developed hypotension and chest pain (Figure 3). Repeat echocardiography was planned to assess the propagation of the dissection, and cardiothoracic surgical consultation was obtained to evaluate the need for coronary artery bypass grafting (CABG). A CT angiogram of the aorta, performed 24 h later, confirmed the absence of retrograde extension into the ascending aorta.

**FIGURE 3:**
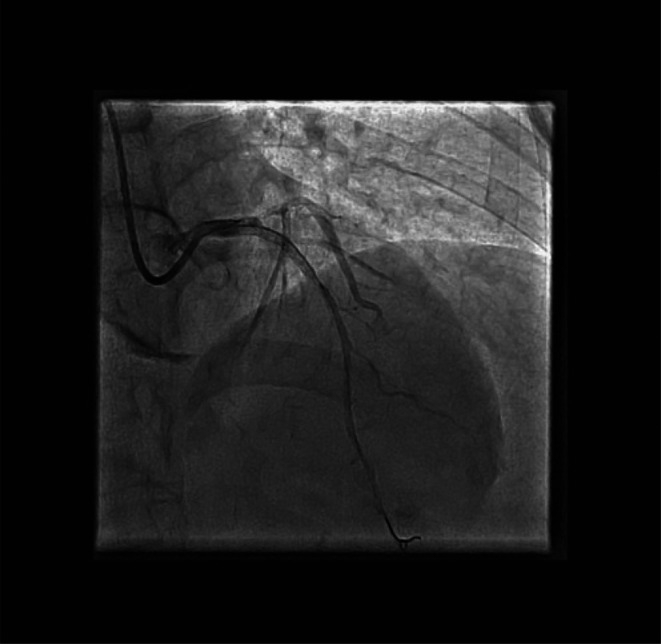
Following the onset of chest pain and a drop in blood pressure, vasopressor therapy was initiated. To seal the dissection entry point and prevent further progression, the stent in the LAD was deployed.

### 3.3 Treatment

Acute haemodynamic instability was managed with vasopressor therapy. The patient was kept nil per os (NPO) and maintained on dual antiplatelet therapy with aspirin and ticagrelor, along with therapeutic anticoagulation using enoxaparin (enoxaparin sodium 60 mg subcutaneously twice daily).

Emergent PCI was subsequently performed to seal the dissection and restore coronary perfusion:

- A 3.0 × 26 mm drug‐eluting stent (DES) was deployed in the LAD to seal the LM dissection entry point (Figure 4), followed by the implantation of a 4.0 × 18 mm DES in the LM (Figure 5)
- Proximal optimisation technique (POT) was performed using a 4.5 × 10 mm noncompliant (NC) balloon in the LM and a 3.5 × 10 mm NC balloon in the LAD (Figure 6)
- Kissing balloon inflation (KBI) was performed using a 3.5 × 10 mm NC balloon in the LAD and a 2.5 × 10 mm semi‐compliant (SC) balloon in the LCx (Figure 7)
- The final POT was completed (Figure 7)

**FIGURE 4:**
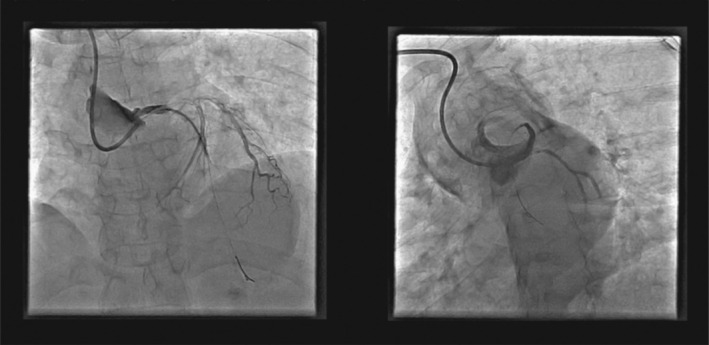
During positioning of a 3.0 × 26 mm DES, severe left main coronary artery dissection was identified, with antegrade extension causing occlusion of the LAD and LCx, and retrograde propagation toward the aortic root and ascending aorta.

**FIGURE 5:**
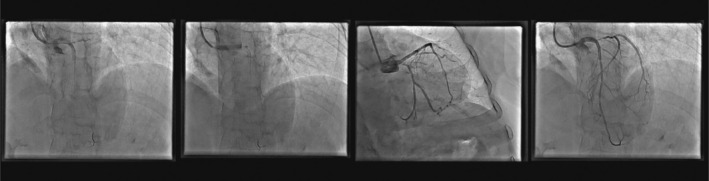
A 4.0 × 18 mm DES was implanted to cover the left main coronary artery. As flow in the LCx was reduced, a coronary guidewire was advanced into the LCx.

**FIGURE 6:**
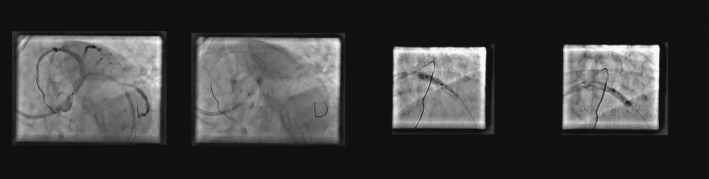
POT and post‐dilation performed using a 4.5 × 10 mm NC balloon in the LM, and a 3.5 × 10 mm NC balloon in the LAD artery.

**FIGURE 7:**
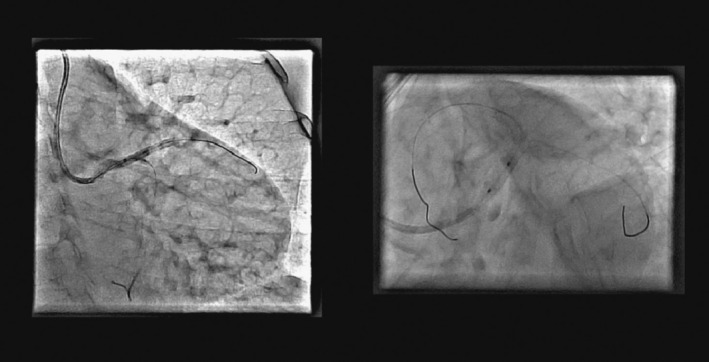
KBI performed to restore LCx flow using a 3.5 × 10 mm NC balloon in the LAD and a 2.5 × 10 mm SC balloon in the LCx, followed by repeat POT.

These interventions successfully restored the coronary flow and sealed the dissection (Figure 8). The patient was then admitted to the coronary intensive care unit (CICU) for close monitoring.

**FIGURE 8:**
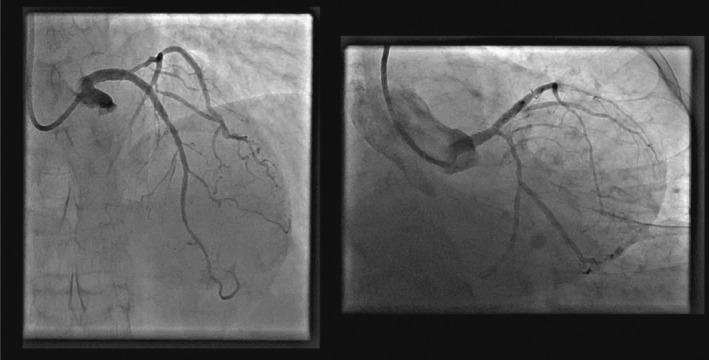
Final results showed restored coronary perfusion with effective sealing of the dissection.

## 4 Conclusion and Results

### 4.1 Outcome

The patient remained haemodynamically stable after the intervention. Follow‐up echocardiography demonstrated no pericardial effusion, aortic regurgitation, or deterioration in ventricular systolic function.

Her CICU stay was uneventful. CT aortography at 24 h post‐procedure corroborated angiographic findings, confirming successful containment of the dissection without extension into the ascending aorta (Figure 9).

**FIGURE 9:**
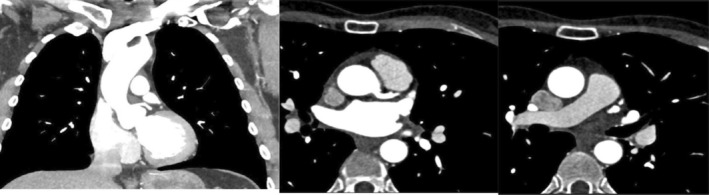
CT angiogram of the aorta confirmed no retrograde extension of the dissection into the ascending aorta, with the dissection fully contained.

### 4.2 Follow‐Up

The patient remained asymptomatic throughout hospitalization and was discharged on guideline‐directed medical therapy, including a statin (atorvastatin), antiplatelet agent (aspirin), P2Y12 receptor blocker (ticagrelor), beta‐blocker (bisoprolol), angiotensin receptor blocker (valsartan), and nitrate (glyceryl trinitrate).

The patient was advised to undergo regular outpatient follow‐up. At the 1‐month follow‐up, she remained symptom‐free, with no recurrence of chest pain or dyspnoea.

## 5 Discussion

Catheter‐induced LMCA dissection is a rare but extremely serious complication of PCI [9]. It can quickly compromise blood flow to a large portion of the myocardium, and if the tear extends into the aortic root, it may also threaten the patient's life through aortic involvement [10]. The reported incidence is low, generally < 0.1% of PCI cases; however, when it occurs, it demands rapid recognition and decisive action [11]. Even more rarely, such iatrogenic dissections can propagate anterogradely or retrogradely into the aortic root, a scenario that poses immediate threats to life and presents unique diagnostic and interventional challenges [12]. Given the rarity of this complication, much of the current knowledge stems from individual case reports, institutional case series and expert consensus.

Several patient‐ and procedure‐related factors predispose to iatrogenic LMCA dissection. Complex coronary anatomy, such as small ostium diameter and/or atheroma, vessel tortuosity, severe calcification, use of larger or deeply seated diagnostic catheters, wedged contrast injection, radial access, forceful advancement of guidewires or balloons, and manipulations in the context of severe atheromatous disease, are risk factors for vessel wall injury and may elevate procedural risk [13]. In our patient, several anatomical and procedural factors increased the technical challenge and likely contributed to the dissection, including non‐coaxial guide catheter seating at the diseased LM ostium via the radial approach, forceful contrast injection against resistance following failed device delivery, and repeated wire and device manipulation through the severely calcified and tortuous LAD, transmitting retrograde mechanical stress to the ostium. The preexisting atherosclerotic burden at the LM–LAD–LCx bifurcation further compromised intimal integrity and lowered the threshold for dissection. The need to navigate a double‐S bend within the LAD further complicates equipment passage and increases the risk of guide catheter or device trauma at the LM ostium. In this context, the “double‐S bend” refers to a morphological description of severe vessel tortuosity in which the LAD follows two successive reverse curves, significantly impeding coaxial device advancement and increasing the risk of guide catheter or equipment‐induced trauma at the LM ostium [14].

The onset of hypotension and chest pain during the procedure was an immediate concern. In such cases, the key priorities are to restore coronary perfusion, seal the entry point of the dissection, and prevent further extension into the aorta [15]. In our case, we achieved rapid sealing using DES in the LAD and LM, supported by POT and KBI. This approach not only reestablished flow but also stabilized the flap and contained retrograde extension. In a similar bifurcation intervention using a bioresorbable vascular scaffold, McGeoch et al. demonstrated that combining the proximal optimisation technique with final kissing balloon inflation facilitated procedural success by optimizing scaffold apposition and maintaining vessel integrity [16]. Notably, when a left‐dominant system is present, as in the current case, a catastrophic event in the LMCA poses a risk to a greater myocardial territory, amplifying the repercussions of delayed recognition and suboptimal management. Close postprocedural monitoring is essential, as dissection extension can occur even after apparent angiographic resolution [17]. In this case, early CT angiography confirmed that the aortic involvement was limited and stable. The patient's uneventful recovery and absence of symptoms at follow‐up highlight that even high‐risk dissections can have good outcomes when managed promptly.

From a broader perspective, this case provides several important lessons for interventional cardiology. First, patient‐ and lesion‐specific planning is critical; highly calcified, tortuous bifurcation lesions in a left‐dominant system may benefit from alternative strategies, such as femoral access or pre‐procedural plaque modification, to reduce device‐delivery difficulties. Second, procedural vigilance and early recognition of subtle signs of dissection can be lifesaving. Finally, a coordinated team approach involving both interventional and surgical teams ensures that all bailout options remain available.

The retrospective consideration of this case also highlights several opportunities for procedural optimisation. Although radial access is now preferred for most PCI because of lower access‐site complications, femoral access with a larger‐bore guiding catheter offers superior passive support and coaxial alignment at the LM ostium in highly complex anatomy, particularly when bulky devices must traverse heavily calcified, tortuous vessels, and should be strongly considered upfront in such cases [18]. Pre‐procedural plaque modification with rotational atherectomy (RA) is another strategy worth considering; RA has demonstrated efficacy in facilitating stent delivery and optimizing acute gain in heavily calcified LM bifurcation lesions, reducing the need for forceful device advancement that risks ostial trauma [19, 20]. Perhaps most critically, intravascular imaging, either intravascular ultrasound (IVUS) or optical coherence tomography (OCT), was not used in this case. IVUS, in particular, carries a Class IA recommendation for guiding LM PCI, providing precise vessel sizing, assessment of calcification severity, guidance on lesion preparation strategy, and early detection of edge dissection or stent malapposition (failure of a stent's metal struts to make full contact with the arterial wall, leaving a gap between the stent and the tissue). Meta‐analytic evidence confirms that IVUS‐guided LM PCI is associated with significantly lower cardiovascular mortality, myocardial infarction, and stent thrombosis than angiography‐guided PCI alone [20, 21, 22]. Incorporating these strategies prospectively in similar high‐risk anatomical settings may reduce the likelihood of iatrogenic complications and improve overall procedural outcomes.

By sharing this experience, we add to the limited literature on catheter‐induced LMCA dissection, particularly in patients with retrograde aortic extension in left‐dominant systems. Such reports are essential for building procedural awareness, refining preventive strategies, and guiding future interventions for similarly complex coronary anatomies.

## Author Contributions

**Samina Yaqoob:** conceptualization, writing – review and editing, writing – original draft. **Ghulam Abbas Shaikh:** conceptualization, supervision, writing – review and editing, writing – original draft. **Zahra Anas:** supervision, writing – review and editing, writing – original draft. **Atiqullah Sadaqat:** writing – original draft, writing – review and editing. **Aniqa Dejwani:** writing – original draft, writing – review and editing. **Ali Aamir:** supervision, writing – review and editing, writing – original draft.

## Funding

The authors have nothing to report.

## Ethics Statement

A written informed consent was obtained from the patient for publication of this case report. All identifying information has been removed or anonymised to protect patient confidentiality.

## Consent

Written informed consent was obtained from the patient for publication and accompanying images. A copy of the written consent is available for review by the Editor‐in‐Chief of this journal on request.

## Conflicts of Interest

The authors declare no conflicts of interest.

## Data Availability

The data that support the findings of this study are available in Pubmed at https://pubmed.ncbi.nlm.nih.gov/, reference number 1. These data were derived from the following resources available in the public domain: Google Scholar, https://scholar.google.com/.
